# 

**Published:** 1894-06

**Authors:** 


### CONTENTS:

Article I.—Some Remarks on the Treatment of Inflamed Pulp. By Frank C. Porter, M. R. C. S., L. R. C. P., L. D. S., D. D. S., Etc. - - - - - - - - 49

" II.—Pyorrhœa Alveolaris. By S. H. Guilford, A.M., D.D.S. 55

" III.—An Act to Amend and Re-enact Sections 1767 and 1774 of the Code of Virginia. - - - - - 58

" IV.—Death From Nitrous-Oxide Gas. By Frank J. Thornbury, M. D. - - - - - - - 62

" V.—Admission to Study Dentistry. - - - - - 65

" VI.—Objections to Amalgam. - - - - - - 69

" VII.—Bridge-Work. By Dr. C. M. Richmond. - - 71

" VIII.—Pericementitis Producing Abscess. By C. N. Johnson, L. D. S., D. D. S. - - - - - - - - 73

" IX.—Combination of Cohesive and Non-Cohesive Gold in Filling. By C. N. Johnson, L. D. S., D. D. S. - 79

### COMMUNICATED.

North-western University Dental School. - - - - - 85

American Dental Association. - - - - - - 86

### EDITORIAL, Etc.

What Dentists are Eligible to Membership in the American Medical Association. - - - - - - - - - - - 86

Southern Dental Association. - - - - - - - 86

A Praiseworthy Effort: A Reference Library for Dentists. - - 87

### MONTHLY SUMMARY.

Swallowed Plates. - - - - - - - - - 87

Aluminum Plates. - - - - - - - - - 88

Killing an Abscess. - - - - - - - - - 89

An Important Decision—The Law Regulating Dentistry in Tennessee Tested. - - - - - - - - - 89

American Dentistry in London. - - - - - - 90

Dental Dots. - - - - - - - - - - 93

How to Get Clean Joints. - - - - - - - 94

Decay of Teeth Coincident With Advanced Civilization. - - 95

Treatment of Broken Impressions. - - - - - - 96

What Dr. T. W. Brophy Says. - - - - - - 96

A New Porcelain. - - - - - - - - - 96

